# Supplementary material for: Sex-specific differences in physiological parameters related to SARS-CoV-2 infections among a national cohort (COVI-GAPP study)
Source: PLoS One. 2024 Mar 6;19(3):e0292203. doi: 10.1371/journal.pone.0292203 (PMC10917257; doi:10.1371/journal.pone.0292203)
Supplement: S1 Table — (DOCX) [file pone.0292203.s001.docx]

Supporting information

**S1 Table. Results from multilevel linear mixed models showing the main effects of infection phase, sex, age, medication, drug and alcohol intake, BMI, and hypertension, as well as interactions between sex and infection phase with regards to changes in physiological signals.**

|  | Skin temperature | Breathing rate | Heart rate | Heart rate variability |
| --- | --- | --- | --- | --- |
| **Intercept** | **35.12 (<0.0001)** | **14.4 (<0.0001)** | **33.86 (<0.0001)** | **4.56 (0.0002)** |
|  |  |  |  |  |
| **Main effects** |  |  |  |  |
| *Infection phase* |  |  |  |  |
| Baseline | Reference | Reference | Reference | Reference |
| Incubation | 0.18 (0.13) | 0.36 (0.09) | 1.63 (0.07) | -0.26 (0.11) |
| Pre-symptomatic | 0.24 (0.24) | 0.75 (0.15) | 1.42 (0.35) | -0.2 (0.39) |
| Symptomatic | **0.73 (<0.0001)** | **2.9 (<0.0001)** | **6.78 (<0.0001)** | **-0.92 (<0.0001)** |
| Recovery | **0.23 (0.0005)** | **0.4 (0.004)** | **2.2 (0.002)** | -0.28 (0.08) |
| *Sex* | **0.44 (<0.0001)** | 0.99 (0.06) | **5.63 (0.0005)** | **-1.33 (<0.0001)** |
| *Age* | 0.002 (0.78) | -0.06 (0.18) | 0.03 (0.8) | 0.02 (0.21) |
| *Medication* | **0.13 (<0.0001)** | **0.32 (<0.0001)** | **0.8 (<0.0001)** | -0.02 (0.65) |
| *Drugs* | 0.13 (0.12) | -0.34 (0.05) | -0.29 (0.66) | 0.03 (0.76) |
| *Alcohol* |  |  |  |  |
| 1-2 alcoholic drinks | -0.01 (0.78) | 0.11 (0.09) | **0.71 (0.008)** | 0.03 (0.53) |
| 3-4 alcoholic drinks | **0.1 (0.006)** | **0.51 (<0.0001)** | **3.25 (<0.0001)** | **-0.15 (0.004)** |
| 5+ alcoholic drinks | **0.12 (0.04)** | **0.77 (<0.0001)** | **5.44 (<0.0001)** | **-0.3 (<0.0001)** |
| *BMI* | -0.007 (0.56) | 0.06 (0.38) | **0.46 (0.03)** | -0.06 (0.06) |
| *Hypertension* | -0.01 (0.92) | 0.21 (0.81) | -4.48 (0.07) | **1.04 (0.009)** |
|  |  |  |  |  |
| **Interactions** |  |  |  |  |
| Sex*Incubation | -0.03 (0.69) | -0.22 (0.07) | -0.5 (0.33) | 0.09 (0.31) |
| Sex*Pre-symptomatic | -0.02 (0.85) | -0.3 (0.32) | -0.06 (0.94) | 0.04 (0.73) |
| Sex*Symptomatic | **-0.28 (<0.0001)** | **-1.32 (<0.0001)** | **-3.12 (0.0001)** | **0.43 (<0.0001)** |
| Sex*Recovery | -0.05 (0.19) | **-0.27 (0.0007)** | **-1.02 (0.01)** | 0.11 (0.24) |

Unstandardized beta coefficients are presented, with p-values in parentheses and in bold if lower than 0.05. Sex was coded such that positive coefficients represent larger values in females. Hypertension was a binary variable representing diagnosed hypertension. In addition to the significant effects of infection phase and sex described in the main text, we observed a significant main effect of medication and alcohol intake on physiological signals. Nevertheless, these effects did not alter any of the multilevel model results reported in the main text.
