# Supplementary material for: Sex-specific differences in physiological parameters related to SARS-CoV-2 infections among a national cohort (COVI-GAPP study)
Source: PLoS One. 2024 Mar 6;19(3):e0292203. doi: 10.1371/journal.pone.0292203 (PMC10917257; doi:10.1371/journal.pone.0292203)
Supplement: Attachment — Submitted filename: Response to Reviewers_20231123.docx [file pone.0292203.s003.docx]

**Response to reviewers**

***PONE-D-23-28587: Sex-specific differences in physiological parameters related to SARS-CoV-2 infections among a national cohort (COVI-GAPP study)***

**Journal requirements**

1. Please ensure that your manuscript meets PLOS ONE's style requirements, including those for file naming.

We made sure that our manuscript meets the PLOS ONE’s style requirements.

2. Thank you for stating the following in the ‘*Competing Interests’* section:

[I have read the journal's policy and the authors of this manuscript have the following competing interests: Lorenz Risch, and Martin Risch are key shareholders of the Dr Risch Medical Laboratory. David Conen has received consulting fees from Roche Diagnostics, outside of the current work. Andjela Markovic, Vladimir Kovacevic and Brianna Goodale are past employees of Ava AG. Billy Franks is a former employee of the Julius Clinic and now employee of Haleon. The other authors have declared that no competing interests exist.].

Please confirm that this does not alter your adherence to all PLOS ONE policies on sharing data and materials, by including the following statement: ""This does not alter our adherence to  PLOS ONE policies on sharing data and materials.” (as detailed online in our guide for authors [http://journals.plos.org/plosone/s/competing-interests](https://urldefense.com/v3/__http:/journals.plos.org/plosone/s/competing-interests__;!!Dc8iu7o!3QqGUvkvvQKk0PAvqcp3BQQXC_uVAu_AO4qMBFdOgNQuuUPbMMo6NciAVUVdKKzXZ0CJad09qcYNaALZsNoxkJSLOk5rcPdAS60oS28$)).  If there are restrictions on sharing of data and/or materials, please state these. Please note that we cannot proceed with consideration of your article until this information has been declared. Please include your updated Competing Interests statement in your cover letter; we will change the online submission form on your behalf.

Thank you for highlighting this. We have followed your proposed adjustments and have changed the section accordingly. As multiple authors of this manuscript have changed their jobs between the time of the study and now, the ‘Competing Interests’ section as stated above needed to be re-written and now reads as stated below:

*‘I have read the journal's policy and the authors of this manuscript have the following competing interests: Lorenz Risch, and Martin Risch are key shareholders of the Dr Risch Medical Laboratory. David Conen has received consulting fees from Roche Diagnostics, outside of the current work. Andjela Markovic, Vladimir Kovacevic, Martina Rothenbühler, Brianna Goodale and Maureen Cronin are past employees of Ava AG. Brianna Goodale and Timo Brakenhoff are current employees of Julius Clinical BV. Billy Franks is a former employee of Julius Clinical BV and now an employee of Haleon. Paul Klaver and Duco Veen are former employees of Julius Clinical BV. Marianna Mitratza is a current employee of P95 CVBA. These competing interests do not alter our adherence to PLOS ONE policies on sharing data and materials.’*

3. In your Data Availability statement, you have not specified where the minimal data set underlying the results described in your manuscript can be found. PLOS defines a study's minimal data set as the underlying data used to reach the conclusions drawn in the manuscript and any additional data required to replicate the reported study findings in their entirety. All PLOS journals require that the minimal data set be made fully available. For more information about our data policy, please see <http://journals.plos.org/plosone/s/data-availability>.

Thank you for highlighting this. The below two sentences have now been added to the revised manuscript in the section ‘Data Availability Statement’:

*‘An anonymized version of the datasets generated in the COVID-RED trial as well as supporting documentation are publicly available through DataverseNL at the following* [*link*](https://dataverse.nl/dataset.xhtml?persistentId=doi:10.34894/FW9PO7) *(doi:10.34894/FW9PO7). Further data that underlie the results reported in our manuscript were collected from study participants from the Principality of Liechtenstein, a very small country, where the risk of subject identification is increased due to the size of the population (less than 40’000 inhabitants). To respect data protection and to prevent the identification of participants, data access is restricted to researchers meeting the criteria for access to confidential data. Data are available from (contact:* [*lorenz.risch@ufl.li.*](mailto:lorenz.risch@ufl.li.%20) [*martin.risch@ksgr.ch*](mailto:martin.risch@ksgr.ch)*, and* [*david.conen@phri.ca*](mailto:david.conen@phri.ca)*).’*

4. Please amend the manuscript submission data (via Edit Submission) to include author Duco Veen.

Author Duco Veen has been added to the manuscript submission data via Edit Submission.

5. One of the noted authors is a consortium [COVID-RED consortium]. In addition to naming the author group, please list the individual authors and affiliations within this group in the acknowledgments section of your manuscript. Please also indicate clearly a lead author for this group along with a contact email address.

The individual authors of the COVID-RED consortium and their affiliations are now added in the acknowledgement section of the revised manuscript. The lead author of this group, Lorenz Risch, along with his contact email address is highlighted as such accordingly.

6. We note that Figure 1 in your submission contain copyrighted images. All PLOS content is published under the Creative Commons Attribution License (CC BY 4.0), which means that the manuscript, images, and Supporting Information files will be freely available online, and any third party is permitted to access, download, copy, distribute, and use these materials in any way, even commercially, with proper attribution. For more information, see our copyright guidelines: <http://journals.plos.org/plosone/s/licenses-and-copyright>.

We require you to either (1) present written permission from the copyright holder to publish these figures specifically under the CC BY 4.0 license, or (2) remove the figures from your submission:
 
A. You may seek permission from the original copyright holder of Figure 1 to publish the content specifically under the CC BY 4.0 license. 
 
We recommend that you contact the original copyright holder with the Content Permission Form (<http://journals.plos.org/plosone/s/file?id=7c09/content-permission-form.pdf>) and the following text:
“I request permission for the open-access journal PLOS ONE to publish XXX under the Creative Commons Attribution License (CCAL) CC BY 4.0 (<http://creativecommons.org/licenses/by/4.0/>). Please be aware that this license allows unrestricted use and distribution, even commercially, by third parties. Please reply and provide explicit written permission to publish XXX under a CC BY license and complete the attached form.”
 
Please upload the completed Content Permission Form or other proof of granted permissions as an ""Other"" file with your submission. 
 
In the figure caption of the copyrighted figure, please include the following text: “Reprinted from [ref] under a CC BY license, with permission from [name of publisher], original copyright [original copyright year].”
 
B. If you are unable to obtain permission from the original copyright holder to publish these figures under the CC BY 4.0 license or if the copyright holder’s requirements are incompatible with the CC BY 4.0 license, please either i) remove the figure or ii) supply a replacement figure that complies with the CC BY 4.0 license. Please check copyright information on all replacement figures and update the figure caption with source information. If applicable, please specify in the figure caption text when a figure is similar but not identical to the original image and is therefore for illustrative purposes only.

As the screenshots in figure 1 have been published in our previous manuscript in BMJ Open (Risch et al., 2022), we now refer to that publication instead of re-using the same figure. The exclusion of figure 1 from this manuscript leads to adjustments in the text, the figures, and the figure legends of figures 1 (previous figure 2) and figure 2 (previous figure 3).

7. Please review your reference list to ensure that it is complete and correct. If you have cited papers that have been retracted, please include the rationale for doing so in the manuscript text, or remove these references and replace them with relevant current references. Any changes to the reference list should be mentioned in the rebuttal letter that accompanies your revised manuscript. If you need to cite a retracted article, indicate the article’s retracted status in the References list and also include a citation and full reference for the retraction notice.

We have added the following four papers to our reference list:

[26] Colantuoni C, Henry G, Zeger S, Pevsner J. Local mean normalization of microarray element signal intensities across an array surface: Quality control and correction of spatially systematic artifacts. Biotechniques. 2002;32: 1316–1320. doi:10.2144/02326mt02

[31] Schaffner, A., Risch, L., Aeschbacher, S., Risch, C., Weber, M. C., Thiel, S. L., ... & Risch, M. (2020). Characterization of a pan-immunoglobulin assay quantifying antibodies directed against the receptor binding domain of the SARS-CoV-2 S1-subunit of the spike protein: a population-based study. *Journal of clinical medicine*, *9*(12), 3989.

[32] Myriam C. Weber, Martin Risch, Sarah L. Thiel, Kirsten Grossmann, Susanne Nigg, Nadia Wohlwend, Thomas Lung, Dorothea Hillmann, Michael Ritzler, Francesca Ferrara, Susanna Bigler, Konrad Egli, Thomas Bodmer, Mauro Imperiali, Yacir Salimi, Felix Fleisch, Alexia Cusini, Sonja Heer, Harald Renz, Matthias Paprotny, Philipp Kohler, Pietro Vernazza, Lorenz Risch, Christian R. Kahlert (2021) Characteristics of Three Different Chemiluminescence Assays for Testing for SARS-CoV-2 Antibodies, Disease Markers, vol. 2021, Article ID 8810196, 13 pages.

[35] Gillespie W. Handbook of the Medical Consequences of Alcohol and Drug Abuse. Paediatr Child Health. 2006;11: 344. doi:10.1093/pch/11.6.344

With these four additional references added to our revised manuscript, our reference list is now complete and correct.

**Editor’s Comments to the Authors**

The paper is a good paper, but may I ask you to submit the row data.

We are thankful for the editor’s comment. As discussed above, the below paragraph has now been added to the section ‘Data Availability Statement’ of our manuscript:

*‘An anonymized version of the datasets as well as supporting documentation generated by the consortium are publicly available through generated in the COVID-RED trial as well as supporting documentation are publicly available through DataverseNL at the following* [*link*](https://dataverse.nl/dataset.xhtml?persistentId=doi:10.34894/FW9PO7) *(doi:10.34894/FW9PO7). Further data that underlie the results reported in our manuscript were collected from study participants from the Principality of Liechtenstein, a very small country, where the risk of subject identification is increased due to the size of the population (less than 40’000 inhabitants). To respect data protection and to prevent the identification of participants, data access is restricted to researchers meeting the criteria for access to confidential data. Data are available from (contact:* [*lorenz.risch@ufl.li.*](mailto:lorenz.risch@ufl.li.%20) [*martin.risch@ksgr.ch*](mailto:martin.risch@ksgr.ch)*, and* [*david.conen@phri.ca)*](mailto:david.conen@phri.ca))*.’*

**Reviewer #1’s Comments to the Authors**

Overall, the study is very well organized and provides a clear and comprehensive overview of the background, research gap, and objectives of the study. The results are clearly stated and discussed. However, I have added a few suggestive comments along with the original manuscript file to be addressed for clarity. I suggested few grammatical and sentence composition suggestions as well using tracked changes, the authors can approve if they find appealing. Please refer to the original file for the comments.

We are very thankful for the suggestive comments added as track changes to the original file. In order to show our answers accordingly, the reviewer’s comments and questions that were directly added to the original manuscript, are listed and commented line by line (see below):

**Line 74**: Add a comma.

A comma has been added.

**Line 87**: Add hyphen.

A hyphen has been added to the corresponding location.

**Line 88**: Add a comma.

A comma has been added.

**Line 91**: Add a comma.

A comma has been added.

**Line 97**: Add ‘such as’.

‘Such as’ has been added to the corresponding location.

**Line 104**: Add hyphen.

A hyphen has been added to the corresponding location.

**Line 118**: Add hyphen.

We refrain from adding a hyphen here.

**Line 119**: Add two hyphens here.

We agree and have added two hyphens to the corresponding location.

**Line 119**: Add a comma.

A comma has been added.

**Line 124**: Add a comma.

A comma has been added.

**Line 125**: Add a comma.

A comma has been added.

**Line 128**: ‘the’ needs to be added.

‘The’ has been added to the corresponding location.

**Line 129**: Add a comma.

A comma has been added.

**Line 134**: Add a comma.

A comma has been added.

**Line 136**: ‘and’ needs to be added.

‘and’ has been added to the corresponding location.

**Line 138**: …goal of assessing and correcting a potential…

We have adjusted this accordingly.

**Line 139:** Overall, the methodology is well-described and provides a strong foundation for the study. However, it might be beneficial to break down some of the longer sentences for improved readability. Consider adding a brief introductory sentence or paragraph that gives a high-level overview of the study design and objectives before delving into specific details. Addressing these comments will further enhance the clarity and completeness of the section.

We thank the reviewer for this suggestion. We have added the following introductory paragraph at the beginning of the methods section to improve readability:

*‘The current study was based on the COVI-GAPP research initiative and included continuous monitoring of biophysical signals by means of a wearable device, the Ava bracelet, coupled with periodic blood tests to assess SARS-CoV-2 antibody titres. Additionally, a ML algorithm was developed based on the COVI-GAPP data to aid in the early detection of COVID-19. This section provides an overview of the methodology employed to address the study’s three primary objectives: 1) investigation of sex differences in COVID-19-related physiological parameters; 2) evaluation of the sex-specific performance of a ML algorithm for early COVID-19 detection; and 3) analysis of sex differences in antibody titres following COVID-19.’*

Additionally, throughout the methods section adjustments have been made to the text to enhance clarity and completeness.

**Line 150: 1**: Specify how compliance with data collection (e.g., bracelet synchronization) was monitored and handled for participants who did not meet the 50% threshold. Provide information on any quality control measures implemented for the physiological data.

We have now added the following sentences: `*Participants were reviewed by a weekly compliance report showing synchronization rates. The study team contacted individuals to follow-up with log-in issues or operational challenges, therefore ensuring quality control.’*

**2:** Consider including a brief statement on data handling, storage, and privacy to address any ethical or regulatory requirements related to participant data.

We agree and have now added the following sentence: `*In order to meet the European Union’s General Data Protection Regulation (GDPR) requirements on participant data, log-in procedure and data handling were performed with an anonymized email account*. `

**Line 155:** Add hyphen.

We refrain from adding a hyphen here.

**Line 157:** Use plural instead of singular.

We agree and have added ‘were’ instead of ‘was’.

**Line 159:** Use plural instead of singular.

We agree and have added ‘bracelets’ instead of ‘bracelet’.

**Line 170**: Please include information on the sensitivity and specificity of the SARS-CoV-2 antibody tests used.

We are thankful for this advice. In order to provide more information regarding the sensitivity and specificity of the antibody tests, the following information including two new references (Schaffner et al., 2020 and Weber et al. 2021; see answers above for full reference) have been added to the corresponding location in the revised manuscript:

*‘[…] N antigen (sensitivity of 96%, specificity of 99.9% for recognition of past SARS-CoV2 infection ) and the receptor binding domain (RBD) of the SARS-CoV-2 spike protein (sensitivity of 97.6%, specificity of 99.8% for recognition of past SARS-CoV2 infection), as described by Schaffner et al. [31] and Weber et al. [32].’*

**Line 180:** Replace ‘as’ with ‘a’.

We refrain from replacing ‘as’ with ‘a’.

**Line 181:** Add a comma.

A comma has been added.

**Line 183:** Replace ‘titel’ by ‘title’.

We agree and have adjusted this accordingly.

**Line 190**: Please clarify if the questionnaire included questions related to comorbidities, as these could potentially influence physiological parameters.

Thank you for this advice. The questionnaire did not include questions related to comorbidities, however, the complementary smartphone app did. The available variables were included in supplementary analyses shown in Table S1. To further clarify this point, we now added the following sentence in the ‘Materials and Methods’ section under ‘Data Collection’:

*‘Furthermore, the app collected information about comorbidities that could potentially influence the physiological signals.’*

**Line 195:** Use singular instead of plural.

We agree and have added ‘weight’ instead of ‘weights.

**Line 196**: Please provide details on the specific potentially confounding variables tested and how they were included in the models.

We have now expanded the description of our sensitivity analysis in the ‘Results’ section as follows:

‘*As a sensitivity analysis, we also tested potentially confounding variables as single terms in additional models to determine whether changes in physiological parameters occurred due to COVID-19 infection over and above changes associated with participant age, BMI, hypertension, medication, alcohol, and recreational drugs. These variables were selected based on previously reported associations with physiological signals [35]. Furthermore, we observed sex differences in BMI and hypertension in the current sample (Table 1) and, therefore, explored in the sensitivity analysis whether these effects can account for the sex differences found in the main analysis. Hypertension, medication and recreational drug intake were binary variables (i.e., yes/no), while alcohol intake was represented through four categories (i.e., none/1-2 drinks/3-4 drinks/5+ drinks* *with none as the reference category). As outlined in the main analysis, four multilevel models were computed (i.e., one for each physiological parameter) additionally including the described variables as fixed effects. In these models, the interactions between sex and phase of infection remained unchanged, indicating that they cannot be explained by the influence of the added variables (S1 Table).’*

**Line 206:** Add comma.

A comma has been added.

**Line 209**: ‘the’ needs to be added.

’the’ has been added to the corresponding location.

**Line 215:** Add hyphen.

A hyphen has been added to the corresponding location.

**Line 216: ‘**the’ needs to be added.

‘the’ has been added to the corresponding location.

**Line 225:** Add comma.

A comma has been added.

**Line 231**: Add comma.

A comma has been added.

**Line 232**: Add ‘from’.

‘from’ has been added to the corresponding location.

**Line 235:** Add ‘the’ twice.

’the’ has been added twice to the corresponding locations.

**Line 236:** Add ‘the’.

‘the’ has been added to the corresponding location.

**Line 237:** Use plural instead of singular.

We refrain from changing from the singular ‘mean’ to the plural ‘means’, because we want to refer to the ‘mean’ (average) and not the ‘means’ as a tool or similar.

**Line 240**: Please consider including a brief explanation of what antibody titres represent and why they are relevant in the context of the study. This will help readers understand the significance of the antibody titre data.

We agree and have now added the following sentence as the final goal of the study in the ‘Introduction’:

*‘Finally, we examined sex differences in antibody levels following COVID-19 to gain additional insights into sex-specific immune responses.’*

Furthermore, we have added the following two sentences in the ‘Materials and Methods’ section: ‘*To gain a deeper understanding of sex-specific differences in the immune system’s reaction to the virus, antibody trajectories were monitored during the study period. Antibody titres reflected the concentration of antibodies in the blood that are specific to the SARS-CoV-2 virus.’*

**Line 241:** Add comma.

We refrain from adding a comma here.

**Line 260:** Add ‘The’ to start the sentence.

We agree and have added ‘The’ to start the sentence.

**Line 269**: It's mentioned that hospitalization data were collected four times (01 – 04). Provide a brief explanation of what these different time points represent and why they were included in the analysis.

We thank the reviewer for pointing out this unclarity. The blood tests were conducted four times over the study period, namely during the following periods:

- June – Nov. 2020 (Blood test 01)
- Dec. 2020 – Feb. 2021 (Blood test 02)
- April – June 2021 (Blood test 03)
- Oct. 2021 – Jan. 2022 (Blood test 04)

During each of these blood tests, information regarding hospitalization was collected from participants. To refer to this point in the manuscript, we have added the following sentences in the ‘Material and Methods’ section under questionnaires:

‘*These visits occurred at approximately 6-month intervals across the duration of data collection.’*

Also, in the caption of Table 1 the following has been added: ‘*Information about hospitalization was collected four times (01 – 04) in the scope of the study centre visit for SARS-CoV-2 antibody tests. It was a part of the questionnaire for SARS-CoV-2 positive participants and represented a measure of disease severity.’*

**Line 269:** Replace ‘were’ by ‘was’.

We’ve replaced ‘were’ with ‘was’.

**Line 276:** Add two commas to this sentence.

We’ve added two commas in this sentence.

**Line 283:** Add comma.

We refrain from adding a comma here.

**Line 296:** Add comma.

A comma has been added to this sentence.

**Line 297:** Add comma.

A comma has been added to this sentence.

**Line 301:** Exchange ‘for’ with ‘of’.

We agree and have added ‘of’ to the corresponding location.

**Line 304:** Add comma.

We refrain from adding a comma here.

**Line 340:** Exchange ‘regards’ with ‘regard’.

We agree and have added ‘regard’ to the corresponding location.

**Line 340:** ‘females’ instead of ‘female’.

We refrain from adjusting this and continue using ‘female’.

**Line 349:** Add hyphen.

A hyphen has been added to the corresponding location.

**Line 357:** Add comma.

A comma has been added.

**Line 366:** Add comma.

A comma has been added.

**Line 368:** Exchange ‘regards’ with ‘regard’.

We agree and have added ‘regard’ to the corresponding location.

**Line 376:** Add comma.

A comma has been added.

**Line 381: ‘**the’ needs to be added.

’the’ has been added to the corresponding location.

**Line 381**: Further Clarification on Menstrual Cycle Effects: While the limitations section acknowledges the potential influence of menstrual cycle-related hormonal changes on physiological parameters, it would be beneficial to include a brief discussion on how this could impact the study's findings. Providing additional context about the potential magnitude of these effects will enhance the comprehensibility of the limitation.

We fully agree with the reviewer that this is an important limitation that needs further discussion. We have expanded our elaboration on this topic as follows:

*‘Particularly during the follicular phase of the menstrual cycle, decreased skin temperature, heart rate and breathing rate have been observed, while heart rate variability was increased. In contrast, the luteal phase was associated with increases in skin temperature, heart rate and breathing rate as well as decreases in heart rate variability, corresponding to the pattern found in COVID-19 patients.’*

*‘Furthermore, hormonal influence offers a plausible explanation only in the first half of the menstrual cycle. The physiological changes observed in its second half could only amplify the trajectory found during COVID-19 in females and thus mask the sex differences in our study. Moreover, the magnitude of physiological changes during COVID-19’s symptomatic phase in the current study is, for all parameters, more than twice as large as the previously reported magnitude of changes across the menstrual cycle [27]. For example, we found that skin temperature increases by 0.7 degrees during the symptomatic phase of COVID-19, whereas this measure’s largest increase during the menstrual cycle is 0.2 degrees during the late luteal phase [27]. Of note, 30% (n=17) of females in the sample were older than 45 years; peri- or post-menopausal, they were beyond natural reproductive age and thus unlikely to experience menses-modulating effects on their physiological parameters. Finally, we do not expect that the distribution of menstrual cycle phases follows a specific pattern for our participants* *(e.g., in complete synchronicity); rather, we expect* *each eumenorrheic woman to* *cycle on her own timeline and the alignment of menstrual phases between participants to occur at random. Taken together, we believe that the hormonal impact on our findings is minimal.’*

**Line 385:** Add comma.

A comma has been added.

**Line 389:** Add comma.

A comma has been added.

**Line 411**: Model Artifacts: The acknowledgment of potential model artifacts in data smoothing is crucial. However, it would be helpful to provide more specific information on steps taken to minimize or account for these artifacts. This could include details about the data preprocessing techniques used.

We appreciate this feedback and agree that providing more specific information on the steps taken to mitigate these artifacts is important for transparency and rigor in our methodology. We have thus included the following additional descriptions in ‘Material and Methods’ section of the revised manuscript:

*‘If a minimum of 4 hours of relatively uninterrupted sleep is achieved, proprietary manufacturer algorithms are employed for pre-processing to eliminate artifacts, identify sleep stages, and provide the nightly physiological parameters. To mitigate potential fluctuations during transitions between wakefulness and sleep, the initial 90 and final 30 minutes of data from each night were excluded. Additionally, each physiological parameter underwent locally estimated scatterplot smoothing (LOESS) before analysis to reduce artificial fluctuations due to measurement errors, aligning with previously established best practices [26]. Further details on the applied data cleaning practices described by the manufacturer can be found in previous publications [7,27].’*

**Line 412**: Adjustment for Multiple Testing: The manuscript correctly points out the lack of adjustment for multiple testing. While it's acknowledged that this could lead to potential type 1 errors, it would be beneficial to suggest potential directions for future research to confirm these initial insights.

We thank the reviewer for this suggestion and have added suggestions for future directions to confirm our insights: *‘Furthermore, upcoming research should explore the mechanisms behind these sex differences, including the roles of sex hormones, genetic factors, and immune responses. Finally, the development of sex-specific treatment strategies, leveraging the insights gained from our study, holds potential for improved patient care and outcomes.’*

**Lines 417 – 419**: The concluding statement regarding the potential of real-time physiological signals for precision medicine is a strong one. However, providing a brief mention of the specific implications for clinical practice or patient care would make the conclusion more actionable for the readers.

We agree that our conclusion may benefit from providing specific implications of our findings for precision medicine. We have extended it as follows: *‘Wearable technology, capable of providing a reliable history of measurements, can empower clinicians with invaluable insights into individual patient health, enabling more personalized and timely interventions that hold promise for improved patient outcomes in the fight against COVID-19 and beyond.’*

**Line 595:** Add two commas to this sentence.

We’ve added two commas to this sentence.

**Line 601:** Add ‘the’ to this sentence.

‘the’ has been added in the corresponding location.

**Line 620:** Add ‘it’ to this sentence.

‘it’ has been added to the corresponding location.

**Reviewer #2 (Dr. Abdurrahman Ahmad El-fulaty)’s Comments to the Authors**

I would suggest removing funding sources from the Abstract section.

We are thankful for this comment and have removed the two sentences about funding sources from the abstract. PLOSONE’s requirements state that funding sources should not be added as acknowledgments or anywhere else in the manuscript file. Our funding information is now only entered in the financial disclosure section of the submission system.

**Table 4, first row:** looking at the mean and SD of 32.66 (±34.74), clearly it doesn't follow the Gaussian distribution. Consider representing the data in median and IQR as the others.

We are thankful for this important comment regarding the first row of table 4. Data for antibody run1 (first row) are now presented as median with IQR.

**Reviewer #3 (Emmanuel Adamolekun)’s Comments to the Authors**

The article followed the right research and Public ethics.

But my question: Is the underlying cardiovascular conditions of the patients play a role in generating Sex-specific differences in physiological parameters relating to the SARS-CoV-2 infections among a research participants? I need some explanation on this.

We thank the reviewer for this comment. We have included the diagnosis of hypertension as a binary variable in our supplementary analysis and found only an association with heart rate variability (S1 Table). However, since the significant sex differences in the physiological changes during COVID-19 persisted, we conclude that they cannot be explained by hypertension. We have additionally expanded our description of this analysis in the result section of our revised manuscript:

‘*As a sensitivity analysis, we also tested potentially confounding variables as single terms in additional models to determine whether changes in physiological parameters occurred due to COVID-19 infection over and above changes associated with participant age, BMI, hypertension, medication, alcohol, and recreational drugs. These variables were selected based on previously reported associations with physiological signals. Furthermore, we observed sex differences in BMI and hypertension in the current sample (Table 1) and, therefore, explored in the sensitivity analysis whether these effects can account for the sex differences found in the main analysis. Hypertension, medication and recreational drug intake were binary variables (i.e., yes/no), while alcohol intake was represented through four categories (i.e., none/1-2 drinks/3-4 drinks/5+ drinks with none as the reference category). As outlined in the main analysis, four multilevel models were computed (i.e., one for each physiological parameter) additionally including the described variables as fixed effects. In these models, the interactions between sex and phase of infection remained unchanged, indicating that they cannot be explained by the influence of the added variables (S1 Table).’*
